# Recent Development toward the Next Clinical Practice of Primary Aldosteronism: A Literature Review

**DOI:** 10.3390/biomedicines9030310

**Published:** 2021-03-17

**Authors:** Yuta Tezuka, Yuto Yamazaki, Yasuhiro Nakamura, Hironobu Sasano, Fumitoshi Satoh

**Affiliations:** 1 Division of Nephrology, Endocrinology and Vascular Medicine, Tohoku University Graduate School of Medicine, Sendai 980-8575, Japan; y.tezuka@med.tohoku.ac.jp; 2 Department of Pathology, Tohoku University Graduate School of Medicine, Sendai 980-8575, Japan; y.yamazaki@patholo2.med.tohoku.ac.jp (Y.Y.); hsasano@patholo2.med.tohoku.ac.jp (H.S.); 3 Division of Pathology, Faculty of Medicine, Tohoku Medical and Pharmaceutical University, Sendai 981-8558, Japan; yasu-naka@patholo2.med.tohoku.ac.jp; 4 Division of Clinical Hypertension, Endocrinology and Metabolism, Tohoku University Graduate School of Medicine, Sendai 980-8575, Japan

**Keywords:** primary aldosteronism, aldosterone-producing adenoma, bilateral hyperaldosteronism, HISTOALDO consensus, steroid profiling, hypertension

## Abstract

For the last seven decades, primary aldosteronism (PA) has been gradually recognized as a leading cause of secondary hypertension harboring increased risks of cardiovascular incidents compared to essential hypertension. Clinically, PA consists of two major subtypes, surgically curable and uncurable phenotypes, determined as unilateral or bilateral PA by adrenal venous sampling. In order to further optimize the treatment, surgery or medications, diagnostic procedures from screening to subtype differentiation is indispensable, while in the general clinical practice, the work-up rate is extremely low even in the patients with refractory hypertension because of the time-consuming and labor-intensive nature of the procedures. Therefore, a novel tool to simplify the diagnostic flow has been recently in enormous demand. In this review, we focus on recent progress in the following clinically important topics of PA: prevalence of PA and its subtypes, newly revealed histopathological classification of aldosterone-producing lesions, novel diagnostic biomarkers and prediction scores. More effective strategy to diagnose PA based on better understanding of its epidemiology and pathology should lead to early detection of PA and could decrease the cardiovascular and renal complications of the patients.

## 1. Introduction

The chronicle of primary aldosteronism (PA) commenced in the 1950s when aldosterone was discovered by Simpson and Tait [1,2]. Conn first clarified the details of PA in a young woman in 1955 [3], following several case reports retrospectively suspected of PA [4,5,6]. After the introduction of the novel syndrome associated with dysregulated aldosterone production, not a few cases with remarkable hypertension and hypokalemia turned out to be PA. Typically, these classic PA cases were detected based on the clinical manifestations of muscle weakness, paresthesia, polyuria, and hypertension without edema [7]. The major cause of PA was experimentally considered as aldosterone-producing adenomas (APAs) [8], while nontumorous etiology of PA was also reported as a surgically uncurable phenotype, termed as “idiopathic hyperaldosteronism” by Liddle [9]. In addition to those sporadic PA cases, Sutherland and the colleagues introduced familial cases of dexamethasone-remediable PA as known as familial hyperaldosteronism (FH) type I [10], further expanding the spectrum of PA. To date, the total of five different types of FH have been established as genetic disorders harboring gene alteration in aldosterone synthase or certain ion channels [11].

For the last seven decades, PA has been gradually recognized as a common hypertensive disorder in contrast to expectation of the low prevalence at the dawn of PA [12,13]. Currently, PA is the leading cause of secondary hypertension, accounting for up to 10% of all hypertensive cases [14]. While accumulating evidence has indicated markedly increased risks of cardiovascular complications and renal dysfunction in PA compared to essential hypertension [15,16,17], efforts to efficiently identify PA and its subtypes preceded development of the diagnostic procedures. For screening, aldosterone-to-renin ratio (ARR) has been the most used indicator for PA since Hiramatsu and the colleagues first demonstrated its utility as a screening tool in 1981 [14,18]. In addition, four confirmatory tests (oral sodium loading, saline infusion, fludrocortisone-suppression, and captopril challenge) have been employed for PA diagnosis [14], based on the assessment of their reliability [19,20,21]. In the discriminative step of PA subtypes, adrenal venous sampling (AVS) is regarded as a gold standard procedure [14], although adrenal imaging partly contributes to the subtype determination [22,23]. In order to systematically detect PA for optimal treatment, clinical guidelines specific to PA have been established based on compelling studies by several organizations of endocrinology [24,25,26,27,28,29,30].

The current diagnostic process for PA includes a number of strict hurdles between screening and subtyping for accurate decision-making on the treatment [31]. However, as large cohorts built the real-world evidence of PA, several issues that need to be reconsidered have been raised, particularly in the path to AVS and the treatment, because of the complexity and physical burden in the diagnostic flow [32,33]. In order to provide a cue for the resolution, numerous studies successively sought to develop novel surrogate markers and prediction scores, particularly for APAs. In this review, we will therefore focus on recent findings about PA, particularly its subtyping, potentially utilized to simplify our diagnostic strategy for PA. In addition, we propose the remaining points for further consideration to achieve rigorous but efficient practice of PA.

## 2. Epidemiology of PA

### 2.1. The Prevalence of PA

The PA prevalence has been estimated as 5–10% in all hypertension based on previous population studies [14], while growing evidence revealed that the real prevalence of PA widely varies depending on several factors such as healthcare levels. Under current guidelines which recommend using ARR in combination with confirmatory tests for PA diagnosis, recent large cohorts in primary care setting have reported that those who have positive screening results for PA accounted for 4.7–25.5% of all hypertensive patients included, and PA was actually detected in 0.7–5.9% of them [34,35,36,37]. In contrast, the PA prevalence increased to 6.0–7.8% in tertiary care centers [38,39]. The differences of PA prevalence between care levels are largely attributed to the different proportion of hypertension stages in addition to their variable screening criteria of plasma aldosterone concentration (PAC) and ARR. Previous studies focusing on severe hypertensive patients have reported the remarkably elevated prevalence of PA, 20.0–29.1% in those who have resistant hypertension [40,41]. Supporting those findings, blood pressure-stratified analysis clearly demonstrated the increase in PA prevalence rates based on the hypertension severity: 3.9% in hypertension stage I, 9.7% in hypertension stage II and 11.8% in hypertension stage III [35].

Intriguingly, a number of case reports have documented biochemically overt PA without hypertension since Brook et al. first described a normotensive PA case with aldosterone-producing carcinoma back in 1972 [42,43]. Several lines of evidence indicated that autonomous aldosterone production that meets the PA confirmatory criteria can be detected in 1.8–14.0% of normotensive population [44,45,46,47]. The detection rates of biochemical PA in normotensive population were lower than in hypertensive population [46,48], while such normotensive PA cases have been also reported to be more likely to develop hypertension within 5 years compared to those without biochemical PA [44]. The clinical significance of asymptomatic PA remains largely unclear, but the subclinical phenotype enables us to consider a continuum model of PA development [47].

### 2.2. Clinical Subtypes of PA

In symptomatic PA, more than 90% of all PA are nonhereditary cases. Those acquired PA consists of two clinical subtypes, unilateral and bilateral forms that the Endocrine Society recommends differentiating by AVS [14]. Unilateral PA is known as a surgically curable case, mostly caused by APAs [49], and bilateral PA, so-called bilateral hyperaldosteronism (BHA) is roughly considered as nontumorous entity which requires mineralocorticoid receptor blocking to abolish the increased cardiovascular risk [50]. The details of pathological classification for aldosterone-producing lesions are summarized in the following section. There are two recent international cohorts, including more than 1000 PA patients who underwent AVS, with emphasis on the subtype prevalence. In the AVIS-2 study collaborating with 19 centers [51], 1302 out of 1625 PA patients who underwent AVS were successfully identified as unilateral (56.8%) or bilateral PA (43.2%) based on the criteria at each local center. In contrast to the dominant prevalence of unilateral PA in the AVIS-2 study, the AVSTAT study consisting of 16 centers reported a lower rate of unilateral identification as 37.0% in PA cases with successful AVS results on the similar basis [52]. Moreover, the authors revealed that regional differences of unilateral PA prevalence (24.0% vs. 47.6% in Japanese and European centers, respectively) and several clinical decisions between these two regions. In addition, a Japanese retrospective cohort suggested age-associated changes of the subtype prevalence in each sex [53]. In men, the unilateral form was more often seen in older PA patients than in younger patients (39% vs. 29%, respectively), whereas the proportion of unilateral PA decreased from 36% to 23% as aging in women. Thus, the prevalence of PA subtypes widely varies depending on age, sex and regional factors.

For PA subtyping, we must also consider the variable procedures in AVS among institutions. The interpretation of AVS results could be influenced by sampling conditions and respective cut-off values for laterality index [54]. Based on those different procedures, Turcu et al. described discordant results of AVS between before and after cosyntropin stimulation [55]. Among 103 cases where adrenal veins were appropriately cannulated, only 69 cases (67.0%) had consistent lateralization of hyperaldosteronism, unilateral or bilateral, between pre- and post-cosyntropin injection, whereas the remaining 34 cases (33.0%) had different lateralization after cosyntropin stimulation compared to that of baseline: from unilateral to bilateral or the opposite change [55]. In addition, a few Japanese institutions have reported an advantage to assess aldosterone concentrations in adrenal tributary veins for precise localization of hyperaldosteronism [56,57,58]. Segmental AVS is a relatively new and challenging technique to detect the exact segment of aldosterone overproduction within an adrenal gland, which can enable us thorough discussion on the pathogenesis and subsequent treatment options, including partial adrenalectomy, in each case. As previously reported, this procedure could provide more appropriate localization in 5.7–19.8% of PA patients compared to conventional AVS [56,57]. Therefore, clinical subtypes of PA, particularly bilateral PA, are considered heterogenous across centers, although histopathological evaluation of the resected adrenal gland can confirm the etiology in PA cases treated by adrenalectomy.

On the other hand, FH is an uncommon form which presents early onset of PA [11]. All subsets of FH are inherited by autosomal dominant pattern. To date, the prevalence has been described as 0.67% in FH type 1, 6.0% in FH type 2 and 0.3% in FH type 3 [59], while the frequencies of other two subsets are still undetermined. The clinical manifestations of FH type I are similar to those of sporadic PA, whereas most cases with FH type I present eukalemia [60]. In addition, intrafamilial variability is observed in the severity of hypertension and its complications, particularly in cerebrovascular events [61,62]. The clinical details of other FH subsets are still unclear due to their rarity. Genetic tests are recommended to detect FH in PA cases with a family history of hypertension and/or child-onset hypertension [63,64]. The genes responsible for FH types I, II, III, IV, and V are *CYP11B2/CYP11B1* chimeric gene, *CLCN2*, *KCNJ5*, *CACNA1H*, and *CACNA1D*, respectively [11].

## 3. Histopathological Classification of PA and Their Expression Profiles of Steroidogenic Enzymes

### 3.1. The HISTALDO Consensus

Various histopathological classifications and nomenclatures have been proposed at this juncture [65,66,67,68]. Recently, international consensus of PA histopathology (HISTALDO) has been published and the nomenclatures were updated and redefined [49]. PA is mainly classified into two major pathological subtypes; neoplastic and non-neoplastic/hyperplastic lesions. In particular, various nomenclatures have been proposed to characterize the non-neoplastic/hyperplastic lesions. In this section, the updated histopathological classification and their immunohistochemical findings are summarized in order to clarify the endocrinological and histopathological characteristics of these updated PA entities. The difference between neoplastic and non-neoplastic lesions were not only detected in their morphological features as well as their expression profiles of steroidogenic enzymes although their differentiation could be occasionally difficult.

**Figure 1:**
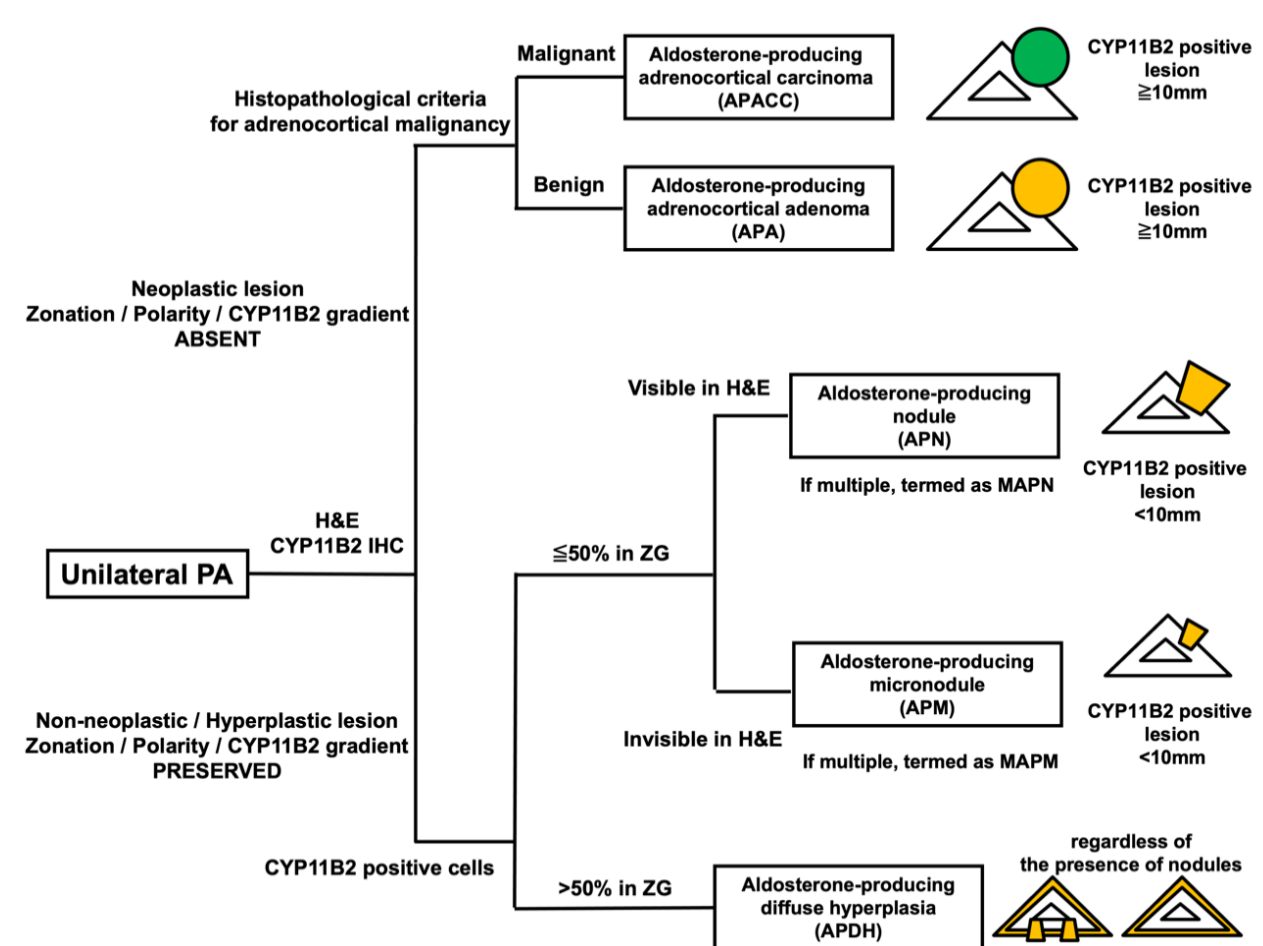
Algorithm of histopathological classification “HISTALDO”. HISTALDO is the classification used for unilateral PA cases [49]. Unilateral PA cases are initially classified into neoplastic or non-neoplastic/hyperplastic lesions based on the polarity or zonation presence which is represented by the gradient of CYP11B1 immunointensity from outer to inner part. Neoplastic lesions are further classified into malignant APACC or benign APA based on the histopathological criteria for adrenocortical malignancy including Weiss criteria. Non-neoplastic/hyperplastic lesions are secondarily subdivided into the group of “nodules” or “diffuse hyperplasia” based on the occupancy of CYP11B2 positive cells in ZG. Nodules are further classified into APN and APM (previously termed as aldosterone-producing cell cluster) whether the nodule could be detected in H&E-stained sections. However, the distinction between APNs and APMs is occasionally difficult and still remained unclear. PA, primary aldosteronism; CYP11B1, 11ß-hydroxylase; APACC, aldosterone-producing adrenocortical carcinoma; APA, aldosterone-producing adenoma; CYP11B2, aldosterone synthase; ZG, zona glomerulosa; (M)APN, (multiple) aldosterone-producing nodule; (M)APM, (multiple) aldosterone-producing micronodule; APDH, aldosterone-producing diffuse hyperplasia; IHC, immunohistochemistry; H&E, hematoxylin and eosin staining.

Based on the statement of HISTALDO (Figure 1) [49], neoplastic lesions were tentatively classified into aldosterone-producing adrenocortical carcinoma (APACC) and aldosterone-producing adenoma according to the histopathological diagnostic criteria for adrenocortical malignancy including Weiss criteria [69]. Non-neoplastic/hyperplastic lesions were classified into focal or diffuse pattern based on the distribution of aldosterone-producing cells examined by immunohistochemistry of aldosterone synthase (CYP11B2) [49,68]. Diffuse pattern was newly termed as “Aldosterone-producing diffuse hyperplasia (APDH)” with the hyperplastic zona glomerulosa (ZG) occupying more than 50% positive for CYP11B2, regardless of the presence of CYP11B2 positive nodules [49]. In contrast, focal patterns were tentatively classified into multiple aldosterone-producing micronodules (MAPMs) or multiple aldosterone-producing nodules (MAPNs) harboring multiple CYP11B2 positive nodules. APMs were previously termed as “aldosterone-producing cell clusters (APCCs) [69,70]” and further defined as the nodules invisible by hematoxylin and eosin (H&E) stained sections located beneath the subcapsular area [49]. On the other hand, aldosterone-producing nodules (APNs) were defined as the non-neoplastic nodules visible by H&E-stained sections some of which are occasionally located distant from the adrenal capsules [49]. The updated classification has made it possible to differentiate between APMs and APNs only by histopathological findings. However, difficult cases have been occasionally associated with differentiating these nodules from each other. The differences between micro-APAs and those non-neoplastic nodules (APMs and APNs) were whether the polarity (zonation) was preserved in the lesions or not [71]. The gradient of CYP11B2 immunointensity from outer to inner part was detected in APMs and APNs, which demonstrated the presence of polarity [71].

These histological entities were defined in unilateral cases, but only few previous studies provided the detailed histopathological findings of bilateral diseases [50,68]. Based on our previous studies, histological entities of bilaterally resected adrenal glands with bilateral diseases revealed the same histological entities in both sides [68]. Yamazaki et al. examined 25 cases with non-neoplastic aldosterone-producing lesions (7: unilateral, 18: bilateral). Among 18 bilateral cases, 12 undergoing bilateral total or partial adrenalectomy represented APDH (seven cases, 58%) or MAPMs (five cases, 42%), and their histological entities were concordant in both resected sides [68]. In addition, Omata et al. examined 15 cases with BHA determined by AVS who underwent unilateral adrenalectomy. Among them, four cases (27%) were diagnosed with APDH, and 11 cases (73%) harbored micro-APA or MAPM [50].

However, the former study included the cohorts with hyperaldosteronism which did not meet the updated current diagnostic criteria of PA, and the latter study examined unilaterally resected adrenals. Therefore, the details of histopathological findings of bilateral adrenal in bilateral disease and their steroidogenic enzymes’ profiles are required for further clarification.

### 3.2. Immunohistochemical Characteristics in Individual Histological Entities

**Figure 2:**
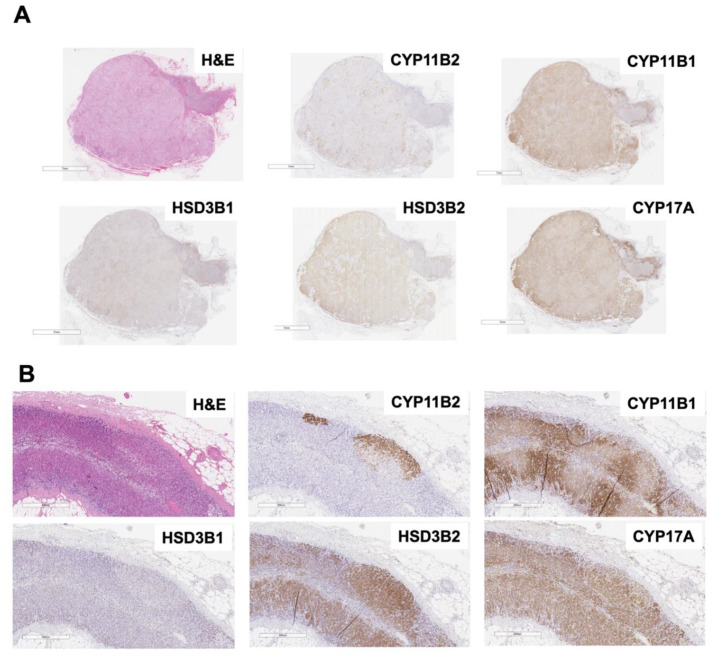
Representative images of an APA and APMs. (**A**). H&E and IHC images of APA. An APA is morphologically composed by clear or compact cells, or mixed pattern. Steroidogenic enzymes involved in in situ aldosterone biosynthesis, such as HSD3Bs and CYP11B2 are immunohistochemically positive, and their immunoreactivity is heterogenous without any disciplined gradients, which suggests the absence of polarity. Enzymes for cortisol biosynthesis are also frequently positive, which is a characteristic of neoplasia. (**B**). H&E and IHC Images of APM. An APM is morphologically composed by clear or compact cells, or mixed pattern. In contrast to APA, polarity is sometimes detected in their morphological features. Steroidogenic enzymes involved in in situ aldosterone biosynthesis, such as HSD3Bs and CYP11B2 are immunohistochemically positive, in particular, immunoreactivity of HSD3B2 and CYP11B2 are concordant in the micronodules. CYP11B2 immunoreactivity shows the characteristic pattern with the gradients from outer to inner, which suggests the presence of polarity. Enzymes for cortisol biosynthesis are generally negative, which is a characteristic of non-neoplastic nature. APA, aldosterone-producing adenoma; APM, aldosterone-producing micronodule; H&E, hematoxylin & eosin staining; IHC, immunohistochemistry; CYP11B2, aldosterone synthase; CYP11B1, 11ß-hydroxylase; HSD3B1 and 2, 3β-hydroxysteroid dehydrogenase type 1 and 2; CYP17A, 17α-hydroxylase.

The different expression profiles of some key steroidogenic enzymes, especially those involved in mineralo- or gluco-corticoids such as 3β-hydroxysteroid dehydrogenase types 1 and 2 (HSD3B1/2), 17α-hydroxylase (CYP17A), 11ß-hydroxylase (CYP11B1), and CYP11B2, could also well-characterize the differences of those histological entities. In addition, different profiles of these enzymes have been also recently reported in APAs with different somatic mutations. Therefore, genotype–immunohistochemical phenotype association is also summarized in this paragraph. Representative histological images are illustrated in Figure 2.

As mentioned above, APMs and APNs preserved their polarity representing that these cells were functionally differentiated into ZG cells although their morphological features did not necessarily resemble ZG cells [68,71]. They expressed the steroidogenic enzymes relevant to in situ aldosterone biosynthesis, such as HSD3Bs and CYP11B2, but not for the enzymes in the different lineage of the adrenocortical zones, such as CYP17A, CYP11B1 [49,67,68,71]. Nakamura et al. reported that CYP11B2 immunoreactivity (H-score) was not different between APAs and non-neoplastic/hyperplastic aldosterone-producing lesions [67]. In contrast, Doi et al. reported that HSD3B1 was specific for ZG cells in BHA, and HSD3B2 was predominantly immunolocalized in the zonae fasciculata and reticularis [72]. Subsequent histological analysis also demonstrated that HSD3B1 immunoreactivity was significantly higher in CYP11B2 negative adjacent ZG intervening between MAPNs, whereas HSD3B2 immunoreactivity was higher in ZG harboring APDH than those adjacent of MAPN [68,73]. Therefore, HSD3B2 immunolocalization could be concordant with CYP11B2 in non-neoplastic aldosterone-producing cells [68], while HSD3B1 could be constitutive although its enzymic activity was more than 5-fold higher than HSD3B2 [74,75]. However, details of their contribution to aldosterone biosynthesis have not been completely unveiled.

On the other hand, aberrant immunolocalization pattern of steroidogenic enzymes was reported to be one of the characteristics of neoplastic aldosterone-producing lesions [71]. In addition, their association with genotypes has been also reported as the characteristic of the disease, which is also different dependent on the ethnicity i.e., *KCNJ5* mutation was detected in more than 70% in East Asian cohorts [76,77,78,79,80], 40% in Caucasian [81], while *CACNA1D* was most frequently detected (≈40%) in Afro-America [82]. Of particular interest, 10–20% of APAs have been reported to complicate with cortisol excess, especially detected in large-sized tumors [83,84]. Ono et al. reported that higher immunoreactivity of steroidogenic enzymes involved in cortisol biosynthesis such as CYP11B1 and CYP17A was more abundantly detected in large-sized tumors [85]. In particular, Tezuka et al. also reported the close association between *KCNJ5* mutation and co-immunolocalization of cortisol-biosynthetic enzymes, especially for CYP11B1 [86]. Hybrid pattern of immunoreactivity of steroidogenic enzymes is one of the characteristics of the neoplastic lesions. In particular, *KCNJ5*-mutated APAs frequently represent large tumors and are composed of abundant clear tumor cells [87,88,89]. Nakamura et al. reported the different types of the hybrid neoplastic cells examined by triple immunofluorescent analysis; CYP11B1+/CYP11B2+, CYP17A+/CYP11B2+ and CYP17A+/CYP11B1+/CYP11B2+ [90]. The population of these hybrid tumor cells was small (CYP11B1+/CYP11B2+, mean 2.1%; CYP17A+/CYP11B2, mean 0.6%; CYP17A+/CYP11B1+/CYP11B2+, mean 0.14%), but the presence of the hybrid tumor cells could be evidence of deviated functional differentiation from the adrenal cortex, which has never been detected in the non-neoplastic/hyperplastic nodules [90]. Different expression profiles dependent on APA genotypes have also been reported. A significantly positive correlation of immunoreactivity was detected between CYP11B2 and CYP17A1 in *KCNJ5*-mutated APAs, while a significantly inverse correlation was detected in *ATP1A1*-mutated APAs [91]. Those differences could indicate the functional differentiation of the tumor cells i.e., *KCNJ5*-mutated APAs were deviated phenotype from ZG cells, whereas *ATP1A1*-mutated APAs were the more differentiating phenotype into ZG cells [91]. Immunolocalization pattern of HSD3Bs in APAs was also reported to be different from APNs/APMs [72,73]. HSD3B2 was the predominant isoform ubiquitously distributed in APAs, while HSD3B1 was heterogeneously detected. Of note, HSD3B1 immunoreactivity was correlated with CYP11B2 in the tumor area, and higher mRNA levels in *KCNJ5*-mutated APAs than wild type [73]. However, detailed expression profiles of HSD3B isoforms in APAs with rare somatic mutations as well as the difference between ethnicity have remained unknown.

## 4. Current Diagnostic Process of PA

### 4.1. Confirmation Process

**Figure 3:**
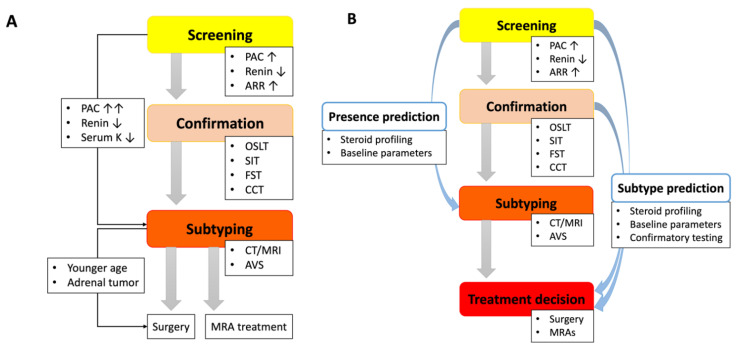
Diagnostic process for PA and potential approach. (**A**). The current diagnostic flow for PA. Currently, PA diagnosis consists of three steps: screening, confirmation and subtyping. After confirmation of PA, therapeutic strategy, surgery or medication, is determined based on the results of AVS. The guideline from the Endocrine Society also mentions specific conditions which allow patients to skip confirmatory tests or AVS. (**B**). Alternative Approach Based on Recent Progress in the Diagnostic Flow. To simplify the diagnostic process, numerous studies have attempted to develop and validate prediction tools using steroid profiles or known clinical parameters. Their approach is composed of two predictive aspects for PA presence and its subtypes. Steroid profiling and scoring systems of baseline characteristics have been reported as useful in both predictions, while modified criteria of confirmatory tests could be also utilized for the subtype differentiation. PA, primary aldosteronism; PAC, plasma aldosterone concentration; ARR, aldosterone-to-renin ratio; OSLT, oral salt loading test; SIT, saline infusion test; FST, fludrocortisone suppression test; CCT, captopril challenge test; CT, computed tomography; MRI, magnetic resonance imaging; AVS, adrenal venous sampling; MRA, mineralocorticoid receptor antagonist.

Here, we focus on the diagnostic process for sporadic PA cases. The Endocrine Society has announced the consensus statements on case detection and subtype differentiation in 2016 [14], followed by agreement of several organizations of endocrinology [26,30,92]. Their concepts include two major goals, (1) to detect cases with inappropriate aldosterone secretion, PA and (2) to determine surgically curable cases, mostly APAs, among the candidates. That is, we can find out hypertensive patients who benefit from treatment with mineralocorticoid receptor antagonists (MRAs) or adrenalectomy through the diagnostic process. In the current guidelines, PA confirmation is composed of two steps, screening and confirmatory tests (Figure 3A). Based on the biochemical features of PA, including autonomous overproduction of aldosterone and subsequent renin suppression, screening for PA is performed with evaluation of both PAC and renin levels and its ratio. For renin measurement, plasma renin activity has been widely used in practice for a long time, while recently, direct renin concentration has come to the fore due to the more rapid and reproducible ability [93,94]. The measurement of plasma aldosterone has also gradually transited from radioimmunoassay to mass spectrometry for the reliability [95,96]. During the screening process, the ARR cut-off value specific to renin assay is applied to narrow down the low-renin cases to those who have a high risk for inappropriate aldosterone secretion. The reliability of the endocrinological evaluation is preserved when the testing conditions are set as under unrestricted salt diet and after correction of hypokalemia and adjustment of medications interfering the renin-angiotensin-aldosterone system (RAAS). Subsequently, those candidates undergo one of any of the following confirmatory tests to prove the presence of PA: oral sodium loading test, saline infusion test (SIT), fludrocortisone-suppression test, and captopril challenge test. Several studies examining the confirmatory testing have reported their reliabilities and similar diagnostic accuracies [21,97,98], although several research groups have also pointed out the discordant results among them [98,99,100]. Of note, a Japanese cohort has demonstrated the interesting advantage of employment of two confirmatory tests in PA confirmation that double-positive results of the testing procedures were associated with high probability of unilateral PA [100].

### 4.2. Subtype Differentiation

Precise determination of PA subtypes allows clinicians to initiate appropriate treatment, surgery or MRAs, leading to better clinical prognosis. As aforementioned, AVS is the recommended procedure to distinguish unilateral PA from bilateral PA, while the details of the protocol, sequential or simultaneous sampling and cosyntropin stimulated or unstimulated setting, are left to each center [14]. The contribution of adrenal imaging to PA subtyping has been also discussed for its less invasive and more feasible nature than AVS. In the middle of the 2000s, a few small studies reported the more accurate lateralization of hyperaldosteronism by AVS than by adrenal imaging (sensitivity, 95% vs. 78%; specificity, 100% vs. 75%, respectively) [101,102]. In contrast, a recent randomized controlled trial, including 200 PA patients seen in European centers, has shown similar clinical and biochemical outcomes after surgical treatment based on adrenal computed tomography (CT) compared to AVS-based treatment [22]. However, using an established outcome scoring system, primary aldosteronism surgical outcome (PASO) criteria [103], an international cohort consisting of 18 centers has demonstrated the decreased likelihood of biochemical remission in PA patients diagnosed by CT compared to the patients diagnosed by AVS [104]. Subsequently, studies comparing the laterality between AVS and adrenal imaging have revealed that the findings of the two procedures were concordant only in 44.6–68.0% of PA patients [105,106,107,108]. Wannachalee et al. has pointed out that the variety of the discordance between AVS and adrenal imaging could result from the differences of race and the prevalence of *KCNJ5*-mutated APAs [109]. Thus, the utility of adrenal imaging alone in subtype differentiation is still controversial. Instead, an adrenal imaging study is regarded as a required step to help the AVS procedure and assess adrenal morphology. Preoperative mapping of adrenal veins, particularly the right side, by CT or magnetic resonance imaging is a vital preparation for successful catheterization in AVS as the adrenal veins are relatively small and have anatomical variation [110,111,112]. Adrenal imaging can be also utilized to evaluate the malignant potential of the adrenal tumor, although APACC is extremely rare [113].

### 4.3. Proposed Bypasses in the PA Diagnostic Flow

In addition to the basic process mentioned above, the diagnostic algorithm provides specific conditions where confirmatory testing and/or AVS could be omitted [14]. Patients with a combination of a high PAC (>20 ng/dL), sufficient renin suppression and spontaneous hypokalemia at screening can directly proceed with the discriminative step by skipping the confirmatory step. This condition is suggested based on the fact that the presence of an elevated PAC and/or hypokalemia reduces false-positive results in ARR rendered by very low renin levels [114,115]. Wang et al. closely examined the diagnostic accuracy of PAC, plasma renin concentration (PRC) and hypokalemia for sparing confirmatory tests by degree [116]. Using the development cohort of 784 patients, they demonstrated that the increase of PAC and PRC was positively and negatively correlated with their specificities for PA diagnosis, respectively, while their sensitivities showed opposite changes [116]. Hypokalemia alone has a specificity of 0.61 and a sensitivity of 0.77 for PA diagnosis, but when combined with PAC (>20 ng/dL) and PRC (<2.5 μIU/mL), the specificity was maximized as 1.00 with a confidence interval of 0.97–1.00. The specificity of this optimized criteria remained 1.00 (a confidence interval, 0.96–1.00) even in the validation cohort, leading to bypassing confirmatory tests in 12% of PA patients [116]. In addition, subtype differentiation can be also circumvented in a case where a young patient has biochemically overt PA with a CT-detectable adrenal tumor. Existing evidence shows that younger age is associated with higher concordance between AVS and CT findings [117,118], allowing us to utilize imaging studies for selection of surgical candidates in a limited situation. In the patients younger than 35 years old with remarkable PA (spontaneous hypokalemia and obvious aldosterone excess) and a solitary adrenal tumor, adrenal imaging could identify unilateral PA with a very high specificity of 0.89–1.00 [119,120,121]. Thus, the current rigorous algorithm for PA diagnosis could be partially spared just in these two conditions.

## 5. Recent Advance in Simplifying the PA Diagnostic Flow

### 5.1. Remaining Issues to Be Considered in the Current Practice

However, the current diagnostic strategy is often considered time-consuming and requires physical burden and expensive cost, particularly in those who undergo AVS [31]. For pretest preparation, medications interfering RAAS should be arranged for hormonal assessment during all the steps. Particularly, use of beta blockers as well as MRAs has a great impact on renin evaluation, resulting in false results of screening and subsequent tests [122,123,124]. On the other hand, in the patients with cardiovascular disease, discontinuing such cardiac protective agents during tests could expose them to a risk for exacerbation. In addition, AVS procedure is occasionally complicated with adrenal hemorrhage, adrenal vein dissection and its infarction, although the complication rates were less than 1% in the specialized referral center [125,126,127]. Finally, completion of the entire flow takes up to a few months using lots of medical resources. Several researchers also raised concern on the impermeability of PA work-up even in patients with severe hypertension. Despite the elevated prevalence of PA, 20.0–29.1% in resistant hypertension, a couple of large cohorts have revealed that only 1.6–2.1% of the patients with resistant hypertension were actually screened for PA [128,129]. Therefore, an alternative approach to PA diagnosis and/or subtype differentiation is unquestionably in great demand not only for efficient identification of PA but also for expansion of the work-up as common management of hypertension.

From the standpoint of clinical subtypes, we could consider several potential approaches which allow us to directly refer the patients for AVS after screening or rapidly induce MRA treatment by skipping the discriminative step (Figure 3B). Theoretically, for those who are eligible for MRA treatment, AVS and/or adrenal imaging can be spared when an alternative method denies the possibility of surgically treatable PA. In contrast, under the present conditions, AVS following adrenal imaging is essential to determine the affected side for surgery in unilateral PA, while confirmatory tests can be omitted if the pretest probability for unilateral PA is sufficiently high at the screening step. To date, numerous studies have attempted to develop a novel tool or reuse an existing test for more effective access to subtype determination and appropriate treatment.

### 5.2. A Novel Insight of Peripheral Steroid Profiling as a Predictive Tool

Steroid dynamics other than aldosterone, particularly 18-hydroxycortisol (18OHF) and 18-oxocortisol (18oxoF), have been investigated to seek the clinical meaning in PA. Mounting evidence revealed the prominent differences in production of 18OHF and 18oxoF, so-called hybrid steroids, between PA and essential hypertension as well as PA subtypes [59,130,131,132]. Following several studies demonstrating the potential of hybrid steroids’ measurement in subtype differentiation, Satoh et al. has proposed a diagnostic flow model for PA incorporating measurement of peripheral 18oxoF in a Japanese PA cohort [133]. In the study, peripheral 18oxoF levels were clearly higher in patients with APAs than in those with BHA, resulting in a high sensitivity and specificity of 0.83 and 0.99, respectively, for discrimination of APAs from BHA at a cut-off value of 4.7 ng/dL [133]. According to their proposed diagnostic model, 18oxoF measurement could allow 43% of BHA patients to start MRA treatment without AVS [133]. Subsequently, Eisenhofer et al. demonstrated a decreased discriminative ability of 18OHF and 18oxoF between APAs and BHA among European PA population, whereas 80% of PA patients were correctly subclassified into the PA subtypes by measurement of 12 steroids employing liquid chromatography–mass spectrometry [134]. Similar results were also reported by an American PA cohort, in which four subgroups based AVS results before and after cosyntropin injection had different steroid profiles in peripheral serum [55]. All those studies indicate that steroid profiling in peripheral blood could circumvent AVS in patients with BHA after PA confirmation, although the used steroids varied among the studies.

Those gaps of steroid concentrations between bilateral and unilateral PA (BHA and APAs) are recognized as a result from unique expression of steroidogenic enzyme in them. As mentioned in the previous section, APAs express various steroidogenic enzymes not only for mineralocorticoids but also glucocorticoids, while other pathological entities of hyperaldosteronism rarely express the enzymes in the different lineage of the adrenocortical zones [67,68,71]. The intra-tumoral environment with CYP11B2 and CYP11B1 coexpressing cells also significantly contributes to enhanced production of hybrid steroids in APAs [86]. Furthermore, APA genotype is also linked with the expression degree of those enzymes, resulting in unique steroid fingerprints by the genotype [91,135]. Williams et al. demonstrated the differences in peripheral steroid concentrations among APA cases with different mutation status [135]. Steroid profiling by liquid chromatography–mass spectrometry revealed that patients with *ATPase*-mutated APAs harbor higher peripheral levels of aldosterone, 11-deoxycorticosterone and corticosterone compared with those with wild-type or *CACNA1D*-mutated APAs [135]. *KCNJ5*-mutated APAs also had distinct features of higher 18oxoF and 18OHF, compared with other APAs [135], probably resulting in a higher discriminative performance of peripheral 18oxoF in Japan than in Europe and US [55,133,134]. The remarkable diagnostic ability of the multi-steroid assessment for *KCNJ5*-mutated APAs represents its impact on steroid profiling as shown in a European cohort [136].

Recently, a European research group has also confirmed the utility of steroid profiling for PA detection. Employing machine learning, a designed steroid panel was validated for prediction of PA with a sensitivity and a specificity of 0.69 and 0.94, respectively [136]. Steroid profiling is, therefore, one of potential predictive tools for both PA confirmation and subtyping. A remaining issue to be discussed is further validation of the steroid panel specific for region and race. Considering the regional and racial characteristics in the prevalence of APA genotypes [80,81,82], optimization of the steroid assessment should be performed at each region. Additionally, reproducibility of the steroid profiles in PA remains unclear. As hormonal measurement is very sensitive to timing, setting and medications, the utility of steroid profiles could be altered by sampling situations. To employ steroid profiling during PA diagnostic process, further examination on steroid dynamics is required.

### 5.3. Modification of the Confirmatory Criteria for Subtype Prediction

Reassessment of the cut-off values for confirmatory tests also brought an additional meaning in subsequent subtyping. With relatively small number of PA patients, a Japanese group revealed another aspect of SIT as a subtyping tool [137]. Based on the analysis of the receiver operating characteristic curve, they proposed an optimal cut-off value of PAC after seated saline infusion as 13.1 ng/dL for subtype prediction, which had a sensitivity of 0.94 and a specificity of 0.79 for unilateral PA [137]. Subsequently, Nagano et al. also examined SIT-based subtype prediction, showing the potential of PAC reduction rate after saline infusion [138]. Using 209 PA cases, they demonstrated that an actual PAC less than 8.79 ng/dL after saline infusion at supine position had a sensitivity and a specificity of 0.80 and 0.86, respectively, for BHA detection, while over 33.8% PAC reduction between before and after SIT had a higher sensitivity of 0.87 [138]. Similarly, Hashimura et al. built a prediction score using both baseline PAC and ARR reduction in SIT with a high specificity of 0.97 for distinguishing BHA, allowing 49% of the cases to circumvent AVS [139]. Thus, combination of different parameters from one confirmatory test could achieve more accurate subtyping than single point measurement of PAC. Comparing with SIT, the captopril challenge and the furosemide upright tests tended to have a slightly lower ability for subtype prediction [138,140].

Besides, several clinical studies have reported that other basic parameters enhance the subtyping ability of SIT. Kocjan et al. conducted a retrospective study to seek a prediction method for BHA [141]. In addition to post-SIT PAC, they selected serum potassium and the presence of CT-detectable adrenal tumors as additional parameters, achieving a specificity of 1.00 for BHA [141]. Additionally, age at diagnosis and baseline renin levels were included in previously reported prediction scores [119,142], whereas the sample size of those studies was not enough for validation. Recently, Burrello et al. conducted a relatively large study to develop and validate prediction models for PA subtyping, using machine learning algorithms [143]. In the study, the researchers included 215 and 118 PA patients, respectively, for the development cohort and the external cohort. Based on the discriminative performance on univariate and multivariate logistic regression analysis, six parameters (baseline PAC, PAC post-confirmatory test, lowest serum potassium, the presence of adrenal nodules and its maximum diameter, and CT findings of adrenal glands) were selected for subsequent analysis, including a linear discriminant analysis model and random forest model [143]. Finally, a 20-point score developed for subtype differentiation, named as SPACE score, demonstrated a high discriminative performance with a maximum accuracy of 89.3% at the score cut-off of 12 in internal validation, whereas the diagnostic accuracy decreased to 78.8% in external validation [143].

Overall, existing evidence indicates that refinement of confirmatory tests, particularly SIT, could benefit PA subtype prediction, incorporating with several basic parameters such as serum. However, as previous studies yielded various criteria and results with relatively small cohorts, a further study with large sample size is still required to establish a solid method. Specific focus on either bilateral or unilateral PA, but not both subtypes, may accelerate the development of the alternative approach to avoid AVS for direct induction of MRA treatment or adrenalectomy, respectively.

### 5.4. Reconsideration of Baseline Characteristics for Prediction of PA

Multiple studies have also attempted to build a prediction score for PA subtypes using baseline characteristics of PA patients [120,121,144,145]. Generally, higher PAC and ARR are associated with unilateral PA cases, while both serum potassium and adrenal appearance on CT are also known as an independent predictor for PA subtypes. Umkoshi et al. pursued the clinical meaning of those parameters in subtype prediction, using 1591 PA patients [118]. In their report, unilateral adrenal abnormality on CT (i.e., a unilateral adrenal tumor) and hypokalemia were strongly associated with the prevalence of unilateral PA cases, which increased from 6.2% in those with normal adrenals and eukalemia to 70.6% in those who had both findings [118]. In agreement with this association, Xiao et al. constructed a clinical nomogram to diagnose BHA cases before AVS, including body mass index, serum potassium and adrenal CT findings [146]. The area under the receiver operating characteristic curve of the nomogram was 0.924 and 0.894 in the training and the validation cohorts, respectively, and the specificity reached to 1.00 when the risk threshold was set as 90% [146]. From another perspective, Puar et al. reported the effectiveness of using a simple tool, plasma aldosterone-to-potassium ratio to predict PA subtypes [147].

Taking advantage of the common availability, a prediction score consisting of baseline parameters could be more practical and useful in PA prediction than subtyping. In the same way as the SPACE score, Burrello et al. constructed a diagnostic scoring system, PACT score, with selected parameters, including sex, antihypertensive medication, serum potassium, the presence of organ damage, renin and aldosterone levels [148]. This 16-point score detected PA patients at internal and external validation with 83.9% and 81.1% of accuracies when the cut-off point was set as equal or greater than 8 [148]. Moreover, in their cohorts, a lower score than 5 completely denied PA, while a score higher than 12 confirmed the PA presence before confirmatory tests [148]. Their proposed algorithm indicates that a total of 22.8% of hypertensive patients could circumvent confirmatory tests and proceed with medication arrangement or AVS [148]. Intriguingly, van Kleef et al. demonstrated the diagnostic performance of their prediction formula in patients with difficult-to-control hypertension to investigate the clinical benefit [149]. The developed formula of predicted probability is composed of seven common parameters, age, systolic blood pressure, serum sodium, serum potassium, potassium supplementation, estimated glomerular filtration ratio, and hemoglobin A1c [149]. Setting the cut-off value as 2.5%, the decision of predicted probability had a sensitivity and a specificity of 0.92 and 0.33, respectively, and 32% of the patients could spare subsequent work-up for PA according to the formula [149].

Based on the easy-to-measure nature and the potential mentioned above, effective use of baseline clinical or biochemical parameters should be further investigated, particularly for skipping the confirmatory step. The prediction model would be cost-effective and could help primary care clinicians with making a decision to refer the patients to tertiary referral centers. Careful consideration of them might also lead to modification of PA screening criteria to increase pretest probability of PA.

## 6. Perspective

Over the past decade after the first guideline for PA management was published by the Endocrine Society, countless studies have successively revealed the epidemiological, pathophysiological and biological characteristics of PA, backed by precise evidence from molecular, genetic and pathological examination. This progress enriched our understanding about PA, allowing us not only to redefine PA itself and pathological entities but also to improve the diagnostic strategy. However, current PA work-up still harbors several issues to be addressed: the time-consuming aspect, patients’ physical burden and the low penetration rate. A recent advance we introduced above accelerates the refinement of the diagnostic process, probably leading to more efficient and easier strategy for PA treatment in the future. Considering the regional and racial differences, further development and validation of alternative approach to subtype differentiation or treatment decision should be thoroughly performed in each PA population. A more sophisticated strategy for PA diagnosis is expected to contribute to early detection and optimization of the treatment, leading to longer healthy life expectancy by decreasing the cardiovascular risk.
